# Delayed diffuse cerebellar swelling after resection of medulloblastoma: case report and review of literature

**DOI:** 10.1007/s00381-017-3496-9

**Published:** 2017-06-29

**Authors:** Ashley L. B. Raghu, Jothy Kandasamy, Mark Brougham, Pasquale Gallo, Drahus Sokol, Mark A. Hughes

**Affiliations:** 1 University of Edinburgh, Edinburgh, UK; 2 Royal Hospital for Sick Children, 9 Sciennes Rd, Edinburgh, EH9 1LF UK; 3 Western General Hospital, University of Edinburgh, Edinburgh, UK; 4 Department of Clinical Neurosciences, Western General Hospital, Edinburgh, EH4 2XU UK; 5 University of Edinburgh, NHS Lothian, Edinburgh, UK

**Keywords:** Medulloblastoma, Delayed cerebellar swelling, Upward herniation, Chemotherapy

## Abstract

**Introduction:**

Delayed diffuse cerebellar swelling is a rare life-threatening complication following medulloblastoma resection.

**Presentation:**

We present our experience of managing a 4-year-old who developed diffuse cerebellar swelling with upward herniation 41 days after resection of a large cell anaplastic medulloblastoma.

**Conclusion:**

Emergency chemotherapy alone was sufficient in promoting regression of swelling and recovery from coma. Reports of similar cases are scant. Chemotherapy may be a critical component of treatment.

## Case report

**Fig. 1:**
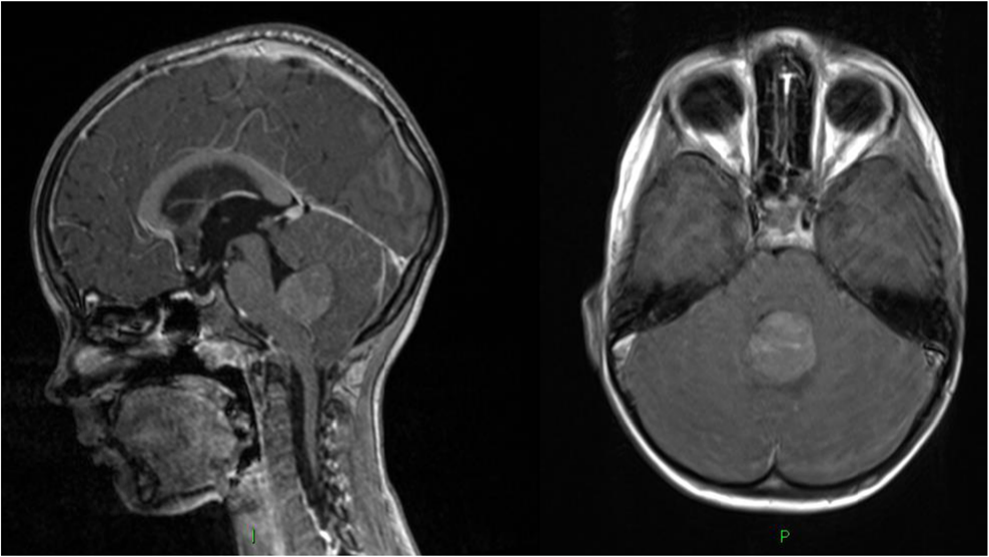
MRI brain at presentation

A 4-year-old boy presented to the emergency department with a two-week history of neck pain, morning vomiting and headaches. He was GCS 15 and had papilloedema. CT and then MRI revealed a solitary 4th ventricular lesion with diffuse leptomeningeal enhancement and early obstructive hydrocephalus (Fig. 1.).

**Fig. 2:**
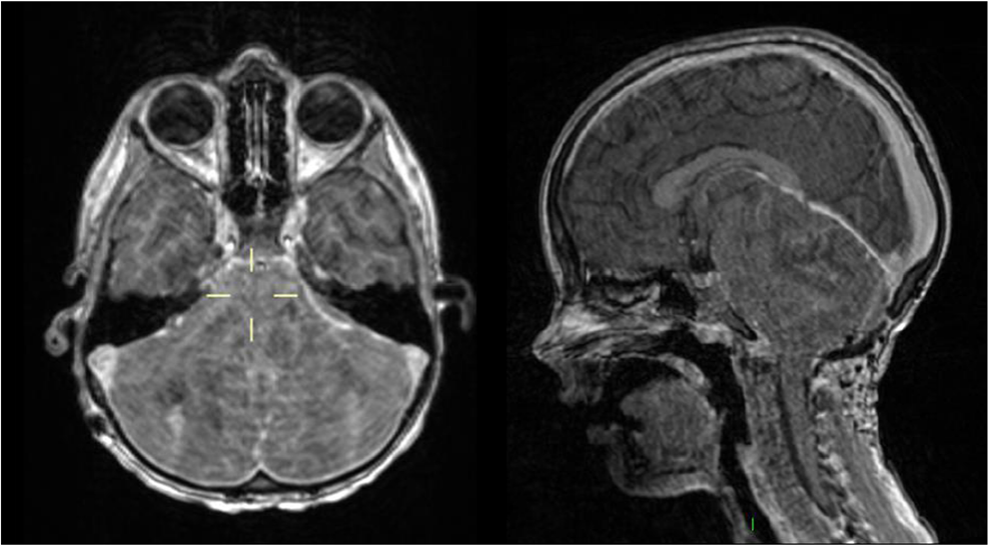
MRI 41 days post-resection

Three days after presentation, he underwent posterior fossa craniotomy for gross total resection of the intra-axial component of the tumour. Histopathological features were consistent with large cell anaplastic (LCA) medulloblastoma, WHO grade IV. Molecular analysis demonstrated Myc amplification. CSF cytology revealed medulloblastoma cells. On the 15th post-operative day, he developed symptoms of hydrocephalus. CT scan was confirmatory and a right parieto-occipital ventriculoperitoneal (VP) shunt was placed. Thirty-three days post-resection, he returned to the hospital for peripheral blood stem cell harvest, the second day of which he became irritable, withdrawn, drowsy and nauseated. CT scan showed hydrocephalus, warranting VP shunt revision. Subsequently, aspiration pneumonia was diagnosed (rhinovirus, *Staphylococcus* *aureus*, *Haemophilus influenzae* grown on secretions). Impaired respiratory function warranted intubation and ventilation for 3 days in ITU, after which he was extubated. However, over the following 3 days he became progressively lethargic, then comatose and then began exhibiting decerebrate posturing. MRI revealed well-decompressed ventricles but diffuse and extensive cerebellar swelling with upward herniation and brainstem compression (Fig. 2.).

**Fig. 3:**
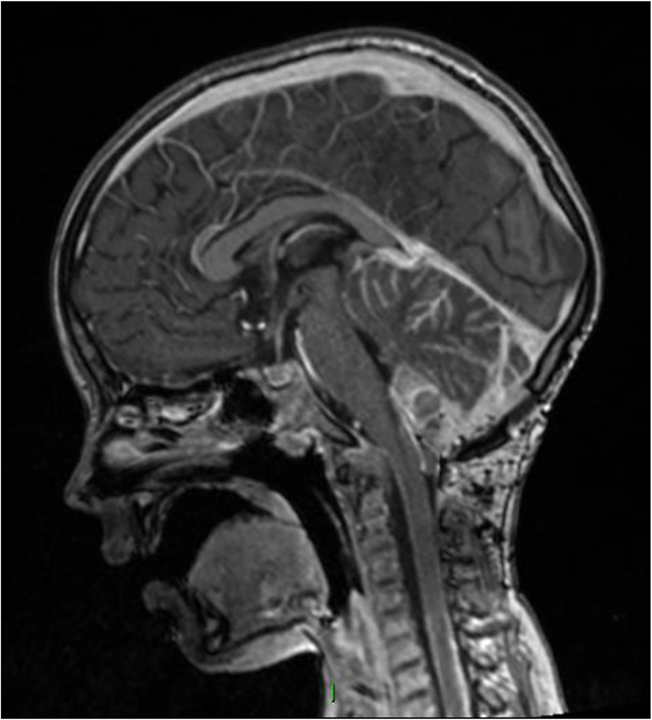
MRI 49 days post-resection

Suspecting that over drainage of supratentorial ventricles may be compounding upward herniation, the VP shunt was externalised and temporarily clamped. However, he deteriorated further, CSF drainage was restarted, and he was re-intubated. Posterior fossa decompression was considered but deemed too high risk given his instability. Emergency chemotherapy with carboplatin and etoposide was commenced, along with high-dose dexamethasone. Three days later, his conscious level was sufficiently improved to allow extubation and chemotherapy was stopped. After a week, he had returned to his baseline post-operative neurological status. MRI performed 7 days after chemotherapy showed resolution of cerebellar swelling (Fig. 3). He went on to receive treatment based on the St Jude’s guidelines for high-risk medulloblastoma [2]. Very sadly, he succumbed to his disease 41 weeks after first presentation.

## Discussion

Only three similar cases are reported in the literature (see Table 1). Ogiwara et al. describe two cases [3]. One patient was managed by posterior fossa decompression alone but failed to respond and died. Another patient was managed initially by posterior fossa decompression and 3 days later chemotherapy was started. Three days after starting chemotherapy (cisplatin, cyclophosphamide, vincristine and etoposide), MRI showed near resolution of oedema with concurrent clinical improvement. The authors recommended early surgical decompression and early chemotherapy. Our case suggests that early rescue chemotherapy alone may be the critical treatment. Shapiro et al. reported a case of midbrain and brain stem oedema which resolved after concurrent radiotherapy and vincristine [4].

**Table 1:** Comparison of four similar cases of delayed cerebellar swelling after resection of medulloblastoma

Authors	Patient Age	Tumour	Metastasis	Oedema	Post-operative HCP	Time to onset	Treatment	Immediate outcome
*Raghu* et al. *2016*	4 years	LCA	Leptomeningeal	Diffuse cerebellar	Yes	41 days	Chemotherapy	Resolution
*Ogiwara* et al. *2010*	31 months	LCA	Leptomeningeal	Diffuse cerebellar	Yes	16 days	Decompression	Failure to respond
*Ogiwara* et al. *2010*	32 months	LCA	Leptomeningeal	Diffuse cerebellar	Yes	35 days	Decompression + chemotherapy	Resolution
*Shapiro* et al. *2011*	3 years	LCA	Leptomeningeal + spinal cord	Midbrain + brainstem	Yes	13 days	Radiotherapy + chemotherapy	Resolution

*LCA* large cell anaplastic medulloblastoma, *HCP* hydrocephalus

The mechanisms underlying delayed diffuse cerebellar swelling in this context are unclear. Its rarity indicates that a number of factors have to align to give rise to the phenomenon. The response to chemotherapy indicates that neoplastic tissue is responsible in some way. Of note, LCA histopathology and presence of leptomeningeal metastasis are common factors in all four cases. Leptomeningeal dissemination may compound perilesional vasogenic oedema through venous drainage obstruction. Tumour-derived vascular permeability factors (VPF) generated by enduring neoplastic tissue may be responsible, but it is unclear why such a crisis would arise late after gross total resection [1]. Transcription switch of VPFs in response to a microenvironment alteration may play a role [5].

The swelling may have been precipitated by an activated immune response co-existing with a dysfunctional blood-brain barrier. Tumour-infiltrating lymphocytes of brain metastases are known to express VPF in correlation with oedema severity [6]. Our patient developed pneumonia prior to the event, and tumour-immune system interplay may be partly culpable. The need for VP shunt was common to all four cases, with shunt revision preceding the development of cerebellar swelling in our case. It is plausible that a change in supratentorial pressure dynamics facilitated upward cerebellar herniation and initiated a cycle of worsening swelling.

## Conclusion

The mechanisms underlying delayed diffuse cerebellar swelling in this context remain unclear. LCA subtype, leptomeningeal disease and concurrent hydrocephalus (with need for VP shunt) appear to be risk factors and should provoke vigilance. Our case, and others, suggests that emergency chemotherapy may be key in promoting regression of swelling and, as such, ought not be delayed.
